# Correlates of exposure to second-hand smoke in an urban Mediterranean population

**DOI:** 10.1186/1471-2458-7-194

**Published:** 2007-08-05

**Authors:** Jorge Twose, Anna Schiaffino, Montse García, Josep Maria Borras, Esteve Fernández

**Affiliations:** 1 Cancer Prevention and Control Unit, Institut Català d'Oncologia, Institut d'Investigació Biomèdica de Bellvitge (IDIBELL), Spain; 2 Department of Experimental and Health Sciences, Universitat Pompeu Fabra, Spain; 3 Organització Catalana de Transplantaments; 4 Department of Clinical Sciences, Universitat de Barcelona, Spain

## Abstract

**Background:**

To describe the socio-demographic factors associated with exposure to second-hand smoke (SHS) in different settings (home, leisure, and workplace).

**Methods:**

We analysed cross-sectional data on self-reported SHS exposure in 1059 non-daily smokers interviewed in the Cornellà Health Interview Survey Follow-up Study in 2002. We calculated age-adjusted prevalence rates and prevalence rate ratios of SHS exposure at home, at the workplace, during leisure time, and in any of these settings.

**Results:**

The age-standardized prevalence rate of SHS exposure in any setting was 69.5% in men and 62.9% in women. Among men, 25.9% reported passive smoking at home, 55.1% during leisure time, and 34.0% at the workplace. Among women, prevalence rates in these settings were 34.1%, 44.3% and 30.1%, respectively. Overall exposure to SHS decreased with age in both men and women. In men, SHS exposure was related to marital status, physical activity, smoking, and alcohol intake. In women, SHS exposure was related to educational level, marital status, occupational status, self-perceived health, smoking-related illness, and alcohol intake.

**Conclusion:**

The prevalence of SHS exposure in this population was high. The strongest association with exposure were found for age and occupational status in men, and age and educational level in women.

## Background

The Surgeon General's report [1] in 1986 and the report of the US Environmental Protection Agency [2] (EPA) in 1992 defined passive smoking or second-hand smoke (SHS) as a health hazard. More recently, SHS has been classified as "carcinogenic to humans (Group 1)" by the International Agency for Research on Cancer [3]. Passive smoking is a proven cause of lung cancer as well as other tumours in non-smokers, and is also related to the development of respiratory diseases in children and adults, and to the appearance of cardiovascular diseases [2-4].

In Spain, where the smoking epidemic was delayed compared to other Western countries[5-9], exposure to SHS was not considered a public health concern until recently [10,11]. Smoking at the workplace and in public places was common and rarely restricted [12-14] until the introduction of a comprehensive anti-smoking law in January 2006. Thus, information on exposure to SHS in the Spanish population is scarce.

Although National Health Interview Surveys have been conducted since 1987, they have included no information on SHS exposure. In Spain the prevalence of exposure to SHS, estimated by self-reported information in an *ad hoc *respiratory survey [15] and local Health Interview Surveys (in the Barcelona area) [16,17], was approximately 60%. These early studies collected information on SHS exposure at home and at the workplace, but not during leisure time, a setting that may be a source of substantial exposure among young people [2,18]. Moreover, little is know about the associations between SHS exposure and socio-demographic characteristics, and both exposure patterns and determinants may be changing due to the implementation in January 2006 of an almost total ban on smoking in enclosed public places and workplaces in Spain [19,20]. We designed the present study [17] to determine the prevalence and determinants of SHS exposure in an urban Mediterranean population in Spain in 2002.

## Methods

### Population

The Cornellà Health Interview Survey Follow-up (CHIS.FU) Study is a prospective cohort study of a representative sample (n = 2500, 1263 women and 1237 men) of the non-institutionalized population of Cornellà de Llobregat, a city located in the metropolitan area of Barcelona, Catalonia (Spain) with a total population of 85061 inhabitants. The design of the CHIS.FU study was described elsewhere [21,22]. Inclusion of the participants in the cohort was based on the date of the interview for the Cornellà Health Interview Survey performed in 1994 (January to December) with face-to-face interviews [23]. In 2002, after record linkage with the municipal census to update vital status and contact information, active telephone follow-up was implemented [24], including a follow-up questionnaire with information on self-perceived health, lifestyles, other health-related variables, and exposure to SHS. Participants gave informed verbal consent to be included both in the 1994 and 2002 interview. All data were managed centrally at the Cancer Prevention and Control Unit of the Catalan Institute of Oncology, following the confidentiality rules for this type of data. The research ethics committee of the Hospital Universitari de Bellvitge/Institut d'Investigacions Biomèdiques de Bellvitge revised and provided ethical approval to the CHIS.FU study.

We obtained a 64.3% response rate in the total cohort: 1608 participants took part in the follow-up interview, with 1438 direct respondents and 170 proxy respondents who answered the questionnaire items on behalf of the index person when he or she was unable to respond because of health problems, or was < 15 years old. There was a 5% refusal rate (n = 123), but 94 of these participants responded to a brief *ad hoc *questionnaire, and thus provided information on educational level, self-perceived health, and smoking behaviour, as well as the reason for declining to participate [21].

Among the 1608 participants interviewed in 2002, we selected those who answered questions about SHS exposure in the follow-up questionnaire. Questionnaires obtained from "proxy" respondents did not include the SHS exposure section of the questionnaire and were thus excluded. We also excluded 379 participants who identified themselves as daily smokers (people who smoked ≥ 1 cigarette/day), since exposure to SHS is irrelevant, but we included those who identified themselves as occasional smokers (< 1 cigarette/day). The final sample analysed here consisted of 1059 participants (449 men and 610 women) aged ≥ 15 years who were never, past or occasional smokers in 2002.

### Dependent variables

The exposure variables were dichotomized as exposure or no exposure to SHS in each of the settings (home, leisure time and workplace, see Appendix). Subsequently a new variable was created: overall self-reported SHS exposure in any of the three settings. From the classification of participants according to the duration of exposure during leisure time, we obtained an approximate estimate of the intensity of exposure. Thus, participants who stated they were not exposed to SHS were coded as "None", those who stated they were exposed during < 1 hour per day from Monday to Thursday or from Friday to Sunday were grouped in the "Low" intensity category; those who were exposed 1 to 4 hours per day were classified in the "Intermediate" intensity category, and participants who were exposed > 4 hours per day on any day of the week were classified int the "high" intensity category. The questionnaire items were derived from previous questionnaires [16] and are currently undergoing validation as part of an ongoing project [25].

### Independent variables

We analysed the following socio-demographic variables: sex, age (15–24 years, 25–44 years, 45–64 years and ≥ 65 years), educational level (≤ primary studies; secondary studies; and university studies), marital status (single, married, or divorced/widowed), and occupational status (employed, unemployed, disabled, retired, housewife, student, or other). We recoded self-perceived health as optimal (very good, good, or fairly good health) and suboptimal (fairly poor, poor or very poor health). Comorbidity was assessed with a list of 16 common chronic conditions and recoded as the absence or presence of any of them, and presence of smoking-related diseases (any of the following: heart disease, varicose veins, asthma, chronic bronchitis, stomach or duodenal ulcer, cerebrovascular disease) was treated as a dichotomous variable (presence or absence of any of these 6 types). We studied physical activity [26,27] as occupational physical activity (inactive, light, moderate or intense) and as leisure physical activity (sedentary, moderate or intense). Smoking was recorded as occasional smoker, former smoker or never smoker; and alcohol intake was recorded as non-drinker, low risk (< 6.87 g/day in men or < 2.75 g/day in women), moderate risk (6.87–17.75 g/day in men or 2.75–8.06 g/day in women), or high risk (> 17.75 g/day in men or > 8.06 g/day in women), according to terciles of consumption in each sex.

### Statistical analysis

We calculated age-specific and age-standardized prevalence rates (and their 95% confidence interval [CI]) of SHS exposure for men and women. Age-standardized prevalence rates were computed by the direct method, using the Cornellà de Llobregat population obtained from the 2001 municipal census as the referent population. We calculated the overall prevalence rate of SHS exposure and the prevalence rates of SHS in different settings, and used a diamond-shaped equiponderant graph [28] to represent the prevalence rates of SHS exposure according to setting in each age group. The plot projects three-dimensional bar graphs in two dimensions whereby the third dimension is replaced with a polygon whose area and middle vertical and horizontal lengths represent the prevalence (%) of participants exposed to SHS.

To evaluate the associations between SHS exposure and different socio-demographic and lifestyle variables, we computed the prevalence rate ratios (PR) and their 95% CI by transformation of the corresponding odds ratios obtained with unconditional logistic regression models [29-31]. Those variables significantly associated in the bivariate analyses were entered in multivariate models for each of the settings (home, leisure time, workplace and overall). All analyses were stratified by sex and adjusted for age.

## Results

**Figure 1:**
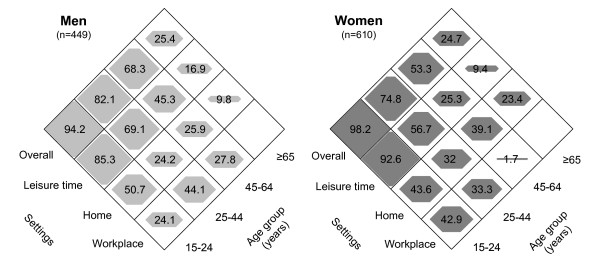
Prevalence (%) of exposure to second-hand smoke among men and women by age group and setting. Cornellà, Spain, 2002.

The age-standardized prevalence rate of self-reported exposure to SHS was 69.5% (95% CI: 64.5%–74.4%) in men and 62.9% (95% CI: 58.1%–67.6%) in women [17]. The prevalence of exposure to SHS differed according to setting (Figure 1): 25.9% (95% CI: 21.8%–30.1%) of the men reported passive smoking at home, 55.1% (95% CI: 50.8%–59.4%) during leisure time, and 34.0% (95% CI: 23.5%–45.6%) at the workplace. In women the prevalence rates for the different settings were 34.1% (95% CI: 29.8%–38.5%), 44.3% (95% CI: 40.5%–48.2%), and 30.1% (95% CI: 18.9%–41.3%), respectively. In both men and women overall self-reported SHS exposure tended to decrease with age, with a significant linear trend (χ^2 ^= 108.9, *P *< 0.01, in men; and χ^2 ^= 126.8, *P *< 0.01, in women, Figure 1). This pattern was also present for SHS exposure during leisure time in men (χ^2 ^= 102.9, *P *< 0.01) and women (χ^2 ^= 163.2, *P *< 0.01), but was less apparent for exposure to SHS at home and at the workplace [17].

**Figure 2:**
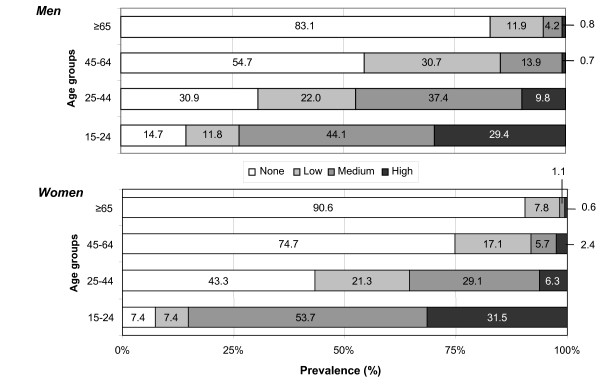
Intensity of exposure (%) to second-hand smoke among men and women during leisure time by age group. Cornellà, Spain, 2002.

The prevalence of high-intensity SHS exposure (more than 4 hours weekly) during leisure time was higher in young people (29.4% in men and 31.5% in women) and decreased to 0.8% in men and 0.6% in women older than 64 years (Figure 2).

The prevalence rate ratios (PR) of exposure to SHS adjusted for age, according to socio-demographic and lifestyle variables, are shown in Table 1 for men and in Table 2 for women. In Tables 3 and 4 we show the results of multivariate analyses for variables that were found to be associated in the bivariate analysis. In men, SHS exposure was associated with occupational physical activity (PR = 1.35; 95% CI: 1.05–1.74 for those who stated being physically active vs. those who were inactive, Table 3). In women, the prevalence of SHS exposure at home was lower among housewives than women employed outside the home (PR = 0.85; 95% CI: 0.72–1.00). Self-reported SHS exposure was higher among women who had any of the six smoking-related diseases (PR = 1.16; 95% CI: 1.02–1.33) (Table 4). In men, SHS exposure during leisure time was related to marital status (lower prevalence in single vs. married men, PR = 0.46; 95% CI: 0.31–0.68), and to high alcohol consumption (PR = 1.40; 95% CI: 1.09–1.81 vs. non-drinkers) (Table 3). In women, SHS exposure was less prevalent in married (PR = 0.59; 95% CI: 0.44–0.78) and divorced or widowed women (PR = 0.75; 95% CI: 0.59–0.95) vs. single women. Exposure to SHS was inversely associated with suboptimal self-perceived health (PR = 0.87; 95% CI: 0.78–0.96) and directly related to moderate or high alcohol intake vs. non-drinkers (Table 4). In men, exposure to SHS at the workplace was associated with occupational physical activity (Table 3). In women, SHS exposure at the workplace was more frequent in those with a university-level education (PR = 1.47; 95% CI: 1.05–2.07 vs. no university education or primary education) and with low alcohol intake (Table 4). Overall SHS exposure in any of the settings was associated in men with being married and being an occasional smoker, whereas in women it was associated with being divorced or widowed, and with no employment outside the home (Tables 3 and 4).

**Table 1:** Age-adjusted prevalence rate ratios (PR) and 95% confidence intervals (CI) of exposure to second-hand smoke in different settings according to socio-demographic variables in men. Cornellà, Spain, 2002.

	**Home **PR (95% CI)	**Leisure time **PR (95% CI)	**Workplace **PR (95% CI)	**Overall **PR (95% CI)
**Educational level**				
≤ Primary studies				
Secondary studies	0.98 (0.77–1.25)	1.07 (0.73–1.55)	1.05 (0.83–1.34)	0.87 (0.53–1.43)
University studies	1.05 (0.85–1.31)	1.00 (0.61–1.63)	0.88 (0.64–1.22)	1.40 (0.65–3.01)
**Marital status**				
Single	1	1	1	1
Married	0.92 (0.78–1.09)	0.49 (0.34–0.71)	0.92 (0.71–1.19)	0.49 (0.28–0.85)
Widowed/Divorced	0.91 (0.69–1.19)	0.63 (0.37–1.05)	1.03 (0.48–2.20)	0.52 (0.27–0.99)
**Occupational status**				
Employed	1	1	-	1
Unemployed	1.18 (0.82–1.71)	1.10 (0.55–2.18)	-	0.66 (0.28–1.57)
Disabled	0.97 (0.76–1.23)	1.06 (0.71–1.59)	-	0.84 (0.46–1.55)
Retired	0.93 (0.81–1.08)	0.94 (0.77–1.14)	-	0.76 (0.55–1.05)
Student	1.10 (0.72–1.67)	0.66 (0.25–1.73)	-	0.80 (0.18–3.49)
Other	0.90 (0.46–1.75)	0.85 (0.26–2.73)	-	0.38 (0.09–1.62)
**Self-perceived health**				
Optimal	1	1	1	1
Suboptimal	1.02 (0.87–1.19)	0.97 (0.78–1.22)	1.06 (0.67–1.68)	0.89 (0.64–1.23)
Comorbidity*				
No	1	1	1	1
Yes	0.99 (0.89–1.10)	1.15 (0.95–1.40)	0.94 (0.77–1.15)	1.05 (0.79–1.40)
Smoking-related diseases**				
No	1	1	1	1
Yes	1.00 (0.90–1.11)	0.87 (0.73–1.05)	0.91 (0.74–1.13)	0.83 (0.64–1.08)
**Occupational physical activity**				
Inactive	1	1	1	1
Light	1.17 (0.94–1.47)	1.24 (0.77–2.00)	1.47 (1.05–2.07)	1.80 (0.86–3.74)
Moderate	1.06 (0.94–1.19)	1.16 (0.93–1.45)	1.08 (0.88–1.33)	1.29 (0.94–1.76)
Intense	1.35 (1.05–1.74)	1.16 (0.71–1.89)	1.45 (1.04–2.01)	2.02 (0.90–4.55)
**Leisure physical activity**				
Sedentary	1	1	1	1
Moderate	0.92 (0.74–1.15)	1.32 (0.94–1.85)	0.72 (0.47–1.11)	1.12 (0.65–1.91)
Intense	0.94 (0.68–1.31)	1.66 (0.95–2.89)	0.79 (0.48–1.31)	1.47 (0.66–3.28)
**Smoking habit**				
Never smoker	1	1	1	1
Occasional smoker	0.99 (0.78–1.26)	1.82 (0.98–3.39)	1.08 (0.74–1.58)	3.56 (1.19–10.67)
Former smoker	0.99 (0.89–1.11)	0.96 (0.80–1.16)	0.87 (0.71–1.07)	0.86 (0.65–1.14)
Alcohol intake***				
Non-drinker	1	1	1	1
Low risk	0.90 (0.76–1.05)	1.19 (0.86–1.64)	1.06 (0.81–1.40)	0.99 (0.63–1.54)
Moderate risk	1.03 (0.89–1.20)	1.20 (0.94–1.55)	0.99 (0.78–1.26)	1.36 (0.93–1.99)
High risk	1.06 (0.91–1.24)	1.36 (1.05–1.76)	1.05 (0.78–1.41)	1.28 (0.89–1.86)

*Absence or presence of chronic diseases

**Absence or presence of smoking-related disease (heart diseases, high blood pressure, varicose veins, asthma or chronic bronchitis, stomach or duodenal ulcer, and cerebrovascular disease).

***Non-drinkers (0 g/day), low risk (< 6.87 g/day), moderate risk (6.87–17.75 g/day) and high risk (> 17.75 g/day)

**Table 2:** Age-adjusted prevalence rate ratios (PR) and 95% confidence intervals (CI) of exposure to second-hand smoke in different settings according to socio-demographic variables in women. Cornellà, Spain, 2002.

	**Home **PR (95% CI)	**Leisure time **PR (95% CI)	**Workplace **PR (95% CI)	**Overall **PR (95% CI)
**Educational level**				
≤ Primary studies				
Secondary studies	0.83 (0.66–1.05)	1.37 (0.94–2.01)	1.14 (0.92–1.43)	1.00 (0.59–1.68)
University studies	0.80 (0.60–1.06)	2.44 (1.40–4.22)	1.51 (1.10–2.08)	6.01 (1.66–21.07)
**Marital status**				
Single	1	1	1	1
Married	0.91 (0.72–1.15)	0.53 (0.41–0.70)	0.93 (0.75–1.16)	0.59 (0.39–0.91)
Widowed/Divorced	1.04 (0.75–1.42)	0.68 (0.54–0.86)	0.94 (0.70–1.27)	0.58 (0.41–0.82)
**Occupational status**				
Employed	1	1	-	1
Unemployed	0.99 (0.69–1.43)	1.19 (0.68–2.08)	-	1.22 (0.49–3.02)
Disabled	0.90 (0.53–1.52)	0.76 (0.52–1.10)	-	0.57 (0.31–1.05)
Retired	0.98 (0.78–1.23)	1.00 (0.88–1.13)	-	0.83 (0.64–1.07)
Housewife	0.84 (0.72–0.99)	0.87 (0.78–0.98)	-	0.64 (0.51–0.80)
Student	1.07 (0.71–1.62)	1.70 (0.45–6.43)	-	1.79 (0.19–16.70)
Other	1.21 (0.67–2.18)	1.02 (0.73–1.43)	-	1.10 (0.57–2.14)
**Self-perceived health**				
Optimal	1	1	1	1
Suboptimal	1.01 (0.85–1.20)	0.82 (0.74–0.92)	1.26 (0.94–1.70)	0.81 (0.68–0.96)
Comorbidity*				
No	1	1	1	1
Yes	1.14 (0.99–1.32)	0.99 (0.86–1.14)	0.94 (0.78–1.12)	1.03 (0.81–1.31)
Smoking-related diseases**				
No	1	1	1	1
Yes	1.17 (1.02–1.33)	1.04 (0.94–1.15)	0.95 (0.81–1.12)	1.13 (0.95–1.35)
**Occupational physical activity**				
Inactive	1	1	1	1
Light	0.98 (0.77–1.24)	1.06 (0.79–1.40)	0.78 (0.58–1.06)	1.06 (0.70–1.60)
Moderate	1.04 (0.88–1.23)	0.99 (0.85–1.15)	0.82 (0.66–1.03)	1.03 (0.81–1.30)
Intense	0.82 (0.60–1.12)	0.82 (0.47–1.42)	0.91 (0.59–1.39)	0.92 (0.45–1.88)
**Leisure physical activity**				
Sedentary	1	1	1	1
Moderate	1.12 (0.86–1.45)	1.05 (0.80–1.38)	0.67 (0.44–1.02)	0.96 (0.64–1.43)
Intense	0.95 (0.73–1.23)	1.03 (0.99–1.07)	0.69 (0.38–1.26)	0.83 (0.49–1.39)
**Smoking habit**				
Never smoker	1	1	1	1
Occasional smoker	0.82 (0.59–1.15)	1.58 (0.76–3.27)	0.72 (0.47–1.10)	1.05 (0.44–2.46)
Former smoker	1.10 (0.91–1.33)	1.12 (0.84–1.47)	1.12 (0.87–1.43)	1.16 (0.78–1.74)
Alcohol intake***				
Non-drinker	1	1	1	1
Low risk	0.99 (0.81–1.19)	1.27 (1.03–1.56)	1.38 (1.07–1.80)	1.35 (0.96–1.91)
Moderate risk	0.93 (0.76–1.14)	1.62 (1.23–2.13)	1.05 (0.85–1.29)	1.23 (0.83–1.81)
High risk	1.10 (0.89–1.37)	1.35 (1.12–1.63)	1.32 (0.99–1.75)	1.41 (1.03–1.93)

*Absence or presence of chronic diseases

**Absence or presence of smoking-related disease (heart diseases, high blood pressure, varicose veins, asthma or chronic bronchitis, stomach or duodenal ulcer and cerebrovascular disease).

***Non-drinkers (0 g/day), low risk (< 2.75 g/day), moderate risk (2.75–8.06 g/day) and high risk (> 8.06 g/day)

**Table 3:** Adjusted prevalence rate ratios (PR)* and 95% confidence intervals (CI) of exposure to second-hand smoke in different settings according socio-demographic variables in men. Cornellà, Spain, 2002.

	**Home **PR (95% CI)	**Leisure time **PR (95% CI)	**Workplace **PR (95% CI)	**Overall **PR (95% CI)
**Marital status**				
Single	-	1	-	1
Married	-	0.46 (0.31–0.68)	-	0.51 (0.29–0.90)
Widowed/Divorced	-	0.62 (0.37–1.05)	-	0.58 (0.31–1.07)
**Occupational physical activity**				
Inactive	1	-	1	-
Light	1.17 (0.94–1.47)	-	1.47 (1.05–2.07)	-
Moderate	1.06 (0.94–1.19)	-	1.08 (0.88–1.33)	-
Intense	1.35 (1.05–1.74)	-	1.45 (1.04–2.01)	-
**Smoking habit**				
Never smoker	-	-	-	1
Occasional smoker	-	-	-	3.47 (1.15–10.44)
Former smoker	-	-	-	0.89 (0.67–1.18)
Alcohol intake**				
Non-drinker	-	1	-	-
Low risk	-	1.21 (0.86–1.69)	-	-
Moderate risk	-	1.26 (0.99–1.62)	-	-
High risk	-	1.40 (1.09–1.81)	-	-

*Adjusted for age and the rest of the variables in the table.

**Non-drinkers (0 g/day), low risk (< 6.87 g/day), moderate risk (6.87–17.75 g/day) and high risk (> 17.75 g/day)

**Table 4:** Adjusted prevalence rate ratios (PR)* and 95% confidence intervals (CI) of exposure to second-hand smoke in different settings according socio-demographic variables in women. Cornellà, Spain, 2002.

	**Home **PR (95% CI)	**Leisure time **PR (95% CI)	**Workplace **PR (95% CI)	**Overall **PR (95% CI)
**Educational level**				
≤ Primary studies				
Secondary studies	-	1.15 (0.74–1.79)	1.16 (0.93–1.45)	0.73 (0.41–1.31)
University studies	-	1.65 (0.83–3.29)	1.47 (1.05–2.07)	3.80 (0.95–15.16)
**Marital status**				
Single	-	1	-	1
Married	-	0.59 (0.44–0.78)	-	0.77 (0.50–1.21)
Widowed/Divorced	-	0.75 (0.59–0.95)	-	0.67 (0.47–0.96)
**Occupational status**				
Employed	1	1	-	1
Unemployed	0.98 (0.68–1.41)	1.13 (0.62–2.09)	-	1.05 (0.41–2.72)
Disabled	0.92 (0.55–1.56)	0.76 (0.51–1.12)	-	0.61 (0.32–1.15)
Retired	0.98 (0.78–1.24)	1.03 (0.91–1.16)	-	0.88 (0.68–1.14)
Housewife	0.85 (0.72–1.00)	0.96 (0.86–1.06)	-	0.65 (0.51–0.82)
Student	1.12 (0.75–1.69)	1.17 (0.25–5.40)	-	1.95 (0.19–19.64)
Other	1.18 (0.64–2.16)	1.12 (0.86–1.46)	-	1.33 (0.74–2.40)
**Self-perceived health**				
Optimal	-	1	-	1
Suboptimal	-	0.87 (0.78–0.96)	-	0.86 (0.72–1.02)
Smoking-related diseases**				
No	1	-	-	-
Yes	1.16 (1.02–1.33)	-	-	-
Alcohol intake***				
Non-drinker	-	1	1	1
Low risk	-	1.21 (0.96–1.52)	1.38 (1.05–1.81)	1.25 (0.87–1.91)
Moderate risk	-	1.58 (1.17–2.13)	1.02 (0.82–1.28)	1.07 (0.69–1.65)
High risk	-	1.30 (1.05–1.61)	1.26 (0.93–1.72)	1.34 (0.95–1.89)

*Adjusted for age and the rest of the variables in the table.

**Absence or presence of smoking-related disease (heart diseases, high blood pressure, varicose veins, asthma or chronic bronchitis, stomach or duodenal ulcer and cerebrovascular disease).

***Non-drinkers (0 g/day), low risk (< 2.75 g/day), moderate risk (2.75–8.06 g/day) and high risk (> 8.06 g/day)

## Discussion

The results of this study show that more than half of the people in the sample were exposed to SHS in 2002. This proportion of passive smokers was similar in both sexes. Previous studies from the same period have found a similar prevalence of SHS exposure in Spain [32,33], although exposure may have changed, especially at the workplaces, after 2006 with the implementation of the new anti-smoking law [34]. Notably, more than 90% of young people (15–24 years old) were exposed to SHS, and approximately 30% of young people reported high levels of exposure.

Our study shows that age was the main determinant of self-reported exposure to SHS: we found a statistically significant inverse association between age and exposure to SHS. Once age was controlled for, marital status was the other main determinant of overall exposure to SHS (single people were more likely to be passive smokers). The association between overall SHS exposure and marital status may be explained by the fact that single persons spend more of their leisure time in places like bars, restaurants or discos, where there were no restrictions on smoking in 2002, (when this study conducted), and where levels of second-hand smoke were likely to be high [35-37].

Other socio-demographic variables such as educational level or occupational status were associated with exposure to SHS at home and at the workplace in women. Educational level among young people was inversely associated with exposure to SHS among women, and directly associated with exposure among men, which is in agreement with current data on smoking prevalence in Spain [38]. In women, we found modest associations between SHS exposure and different health-related variables. Participants who perceived their health as optimal had high leisure-time SHS exposures. It is reasonable to think that healthy people are more likely to spend their leisure time at hospitality venues. However, women who had smoking-related diseases presented a high prevalence of SHS exposure at home. A possible explanation is that these women live with smokers (a factor not investigated in this study), which could lead to high exposures to SHS and thus make smoking-related illnesses more likely. However, the cross-sectional design of the study does not allow us to establish any causal connection.

Occupational physical activity was directly associated with SHS exposure in men at home and at the workplace. Those participants whose job required intense physical effort may work in settings where smoking is frequent, such as the manufacturing or construction sectors [27], so they might work near smokers [39]. In addition, participants from lower social classes, in which the prevalence of active smoking is higher [38,40-42], are more likely to have jobs involving intense physical activity. Previous studies have shown a similar socio-economic gradient in SHS exposure [43,44]. Occasional smoking was also associated with SHS exposure in men, possibly because they interacted with smokers or often went to places where exposure to tobacco smoke was high. Finally, alcohol consumption in both men and women was also associated with SHS exposure, possibly because in 2002 there were no restrictions on smoking in places where alcohol consumption is usual in Spain (mainly bars and restaurants. i.e., enclosed areas where ventilation is less than optimum).

The results of this study show that the prevalence of SHS exposure was high at workplaces and during leisure time in 2002, and that this especially affected young and middle-aged people. This leisure environment is at the same time the workplace for many other people, e.g., employees in the hospitality sector. These workers had worse respiratory health and were more exposed to SHS than participants who lived with a smoker [45], and levels of SHS in restaurants were twofold those in office workplaces where smoking was allowed [37,46,47]. However, the recent introduction of anti-smoking laws has led to a reduction in exposure to SHS in hospitality workers [48-54].

The limitations of this study derive from the fact that cross-sectional data based on information gathered by questionnaire are potentially subject to some degree of systematic error. The method used here to measure SHS exposure has not been formally validated. The questions used in the survey are partially derived from those used in other studies[16], although we added new items designed to enquire about exposure during leisure time [24]. Additional studies on SHS exposure should consider the use of biological markers of passive exposure [55-57]. These limitations notwithstanding, the measurement of "perceived" SHS exposure with a set of simple questions may provide sufficiently valid estimates in the absence of expensive biomarker data [58-62].

Some selection bias was possible because the sample studied was not completely representative of the Cornellà population. The sample we analysed is missing a portion of young and old participants of both sexes, mainly due to attrition attributable to deaths and emigration [22]. Thus, the prevalence of general exposure to SHS might be still higher; since young people (under-represented in the sample) are those who are most exposed.

The estimates of SHS exposure discussed here did not consider duration of exposure, except for leisure time exposure. Although the prevalence of SHS exposure during leisure time is important, its duration is shorter than SHS exposure at home or at the workplace. Unfortunately, we did not collect information on the proportion of time spent in each setting. Doing so would have helped to better understand the contribution of each setting to overall exposure and related risk. Future studies should consider the contribution of each setting to overall exposure and the dose of exposure in more detail, to better quantify the hazard that SHS exposure represents. A final consideration is that we assessed "current" self-reported exposure to SHS, whereas other studies have analysed cumulative lifetime exposure, which is relevant for etiologic studies [63-66].

## Conclusion

The prevalence of self-reported SHS exposure in this Mediterranean population was very high, and correlated mainly with age and occupational status in men, and with age and educational level in women. These findings highlight the need to curtail the smoking epidemic and limit second-hand smoke exposure in Spain. The Spanish law banning smoking in all enclosed workplaces and public places (with certain exceptions for bars and restaurants) is a necessary but insufficient stimulus to achieve smoke-free environments [20,67].

## Abbreviations

SHS: second-hand smoke; CHIS.FU: Cornellà Health Interview Survey Follow-up; CI: confidence interval; PR: prevalence rate ratio.

## Competing interests

The author(s) declare that they have no competing interests.

## Authors' contributions

EF, AS, MG, and JMB designed the study protocol. AS and MG co-ordinated the follow-up data collection. JT, AS and EF designed the study on second-hand smoke. JT checked the information referring to second-hand smoke. JT, AS and EF developed the strategy for analysis, JT performed the statistical analysis, and all the authors contributed to the interpretation of the results. JT wrote the first draft of the manuscript, to which all the authors made contributions. EF is the guarantor of the paper.

## Appendix. Details on questionnaire wording and construction of variables

### Exposure at home

The questions was, "Do any members of your family usually smoke at home?"

### Exposure during leisure time

The question was, "On average, how long are you exposed to tobacco smoke outside your home and at your workplace per day on Monday to Thursday or Wednesday to Sunday?" Duration of exposure was recorded on an ordinal scale as 0, < 1 hour, 1–4 hours, and > 4 hours);

### Exposure at the workplace

The question was, "Do any people smoke near you at the workplace?"

### Exposure at any of the settings (overall second-hand smoke exposure)

Participants who responded that they were not exposed at home, during leisure time or at the workplace were considered "not exposed overall to SHS". Participants who responded affirmatively to any of the questions were considered "exposed overall to SHS".

## Pre-publication history

The pre-publication history for this paper can be accessed here:


